# Testing the Outcomes of a Smoking Cessation Smartphone App for Nondaily Smokers: Protocol for a Proof-of-concept Randomized Controlled Trial

**DOI:** 10.2196/40867

**Published:** 2023-02-14

**Authors:** Bettina B Hoeppner, Kaitlyn R Siegel, Sarah R Dickerman, Akshiti A Todi, Christopher W Kahler, Elyse R Park, Susanne S Hoeppner

**Affiliations:** 1 Recovery Research Institute Department of Psychiatry, Massachusetts General Hospital Harvard Medical School Boston, MA United States; 2 Biobehavioral Laboratory Department of Psychiatry, Boston Children's Hospital Harvard Medical School Boston, MA United States; 3 Department of Psychiatry University of Oxford Oxford United Kingdom; 4 Center for Alcohol and Addiction Studies Department of Behavioral and Social Sciences Brown University School of Public Health Providence, RI United States; 5 Mongan Institute Department of Psychiatry, Massachusetts General Hospital Harvard Medical School Boston, MA United States; 6 OCD & Related Disorders Program Department of Psychiatry, Massachusetts General Hospital Harvard Medical School Boston, MA United States

**Keywords:** mobile health, mHealth, smartphone, smartphone app, smoking, smoking cessation, nondaily smoking, positive psychology, happiness, positive affect, clinical trial, feasibility, acceptability, app use, mobile phone

## Abstract

**Background:**

Nondaily smoking is a widespread, increasingly prevalent pattern of smoking, particularly in ethnic minority and vulnerable populations. To date, no effective treatment approach for this type of smokers has been identified.

**Objective:**

This study aims to use a randomized controlled trial to evaluate proof-of-concept markers of the Smiling instead of Smoking (SiS) app, a smoking cessation smartphone app designed specifically for nondaily smokers. This app was developed iteratively and is now in its third version. Previous studies have demonstrated acceptability and feasibility when participants were onboarded in person (study 1) and remotely (study 2) and showed within-person changes in line with hypothesized mechanisms of change. This is the first randomized test of this app.

**Methods:**

In total, 225 adult nondaily smokers will be asked to undertake a quit attempt while using smoking cessation support materials for a period of 7 weeks. Participants will be randomized to use the SiS smartphone app, the National Cancer Institute smartphone app QuitGuide, or the National Cancer Institute smoking cessation brochure “Clearing the Air.” Participants will take part in a 15-minute scripted onboarding phone call during which study staff will introduce participants to their support materials. Survey links will be sent 2, 6, 12, and 24 weeks after the participants’ initially chosen quit date. The primary outcome is self-efficacy to remain abstinent from smoking at treatment end, measured using the Smoking Self-Efficacy Questionnaire. Secondary outcomes cover several domains relevant to treatment development and implementation: treatment acceptability (eg, satisfaction with smoking cessation support, measured using the Client Satisfaction Questionnaire, and app usability, measured using the System Usability Scale); treatment feasibility (eg, measured using the number of days participants used the SiS or QuitGuide app during the prescribed treatment period); and, in an exploratory way, treatment efficacy assessed using self-reported 30-day point prevalence abstinence.

**Results:**

Recruitment began in January 2021 and ended June 2022. The final 24-week follow-up was completed in January 2023. This trial is funded by the American Cancer Society.

**Conclusions:**

This study is designed to test whether the prescribed use of the SiS app results in greater self-efficacy to abstain from smoking in nondaily smokers than commonly recommended alternative treatments and whether the SiS app treatment is acceptable and feasible. Positive results will mean that the SiS app warrants testing in a large-scale randomized controlled trial to test its effectiveness in supporting smoking cessation in nondaily smokers. The design of this study also provides insights into issues pertinent to smoking cessation smartphone app treatment development and implementation by measuring, in a randomized design, markers of treatment satisfaction, engagement with the technology and content of the treatment, and adherence to the treatment plan.

**Trial Registration:**

ClinicalTrials.gov NCT04672239; https://clinicaltrials.gov/ct2/show/NCT04672239

**International Registered Report Identifier (IRRID):**

DERR1-10.2196/40867

## Introduction

### Background

Cigarette smoking remains the number 1 cause of preventable disease and death in the United States, responsible for 16 million current cases of smoking-related diseases [1]. Although the prevalence of smoking has steadily declined over the past few decades, in part because of expansive public health efforts, taxation and indoor smoking policies, and accessibility to evidence-based cessation tools, the prevalence of nondaily smoking is steadily increasing. Currently, 25.4% of all adult smokers are nondaily smokers, a jump from 20.2% in 2008 [2]. Nondaily smoking has been a recognized public health issue for >10 years [3], yet the knowledge gap about treating nondaily smokers has remained largely unchanged, presenting a significant barrier to supporting this growing population of smokers in their cessation efforts [4]. However, knowledge has been gained that underscores the urgency of addressing nondaily smoking.

First, the detrimental health impact of nondaily smoking has now been clearly demonstrated. Nationally representative data have shown that, despite smoking a low number of cigarettes per month, nondaily smokers are exposed to substantial concentrations of carcinogenic nitrosamines [5]. Individuals who smoke as little as 6 to 10 cigarettes per month have a higher all-cause mortality risk than never smokers [6]. For individuals who smoke 11 to 30 cigarettes per month, the mortality risk compared with never smokers increases by 72%, and the median life expectancy decreases by 5 years [7]. Nondaily smokers are also at greater risk of cancer, heart disease, and respiratory disease [6].

Second, nondaily smoking is disproportionally prevalent in disadvantaged and vulnerable groups. Nondaily smoking is consistently highest among non-Hispanic Black individuals [8,9]. Nondaily smoking is also increasingly prevalent among adults with mental health and substance use problems [10], 2 groups with substantially higher smoking prevalence rates (35.8% in adults with serious psychological distress [11] and 65%-87% in individuals with substance use disorders [12]) than the general population (15.5%) [11]. Thus, failure to address nondaily smoking contributes to and further widens existing health disparities.

A promising method of engaging and meeting the needs of nondaily smokers are smartphone apps. Nondaily smokers are motivated to quit smoking, more so than daily smokers [13-15], with more recent and planned cessation efforts [14,16-18]. However, similar to daily smokers, nondaily smokers frequently fail in their quit attempts [19], demonstrating their need for smoking cessation support. Traditional treatment options fail to appeal to nondaily smokers, as evidenced by their lower rates of seeking or receiving formal smoking cessation treatment [13,14,20,21]. For nondaily smokers, many of whom resist labeling themselves as *smokers* [22-24], smartphone apps represent a low-barrier method for receiving smoking cessation support that invokes less treatment resistance than more traditional smoking cessation treatments [20].

Smartphone apps for smoking cessation have been increasingly prolific in recent years. Currently, there are an estimated 490 English-language smoking cessation apps on the market, downloaded an estimated 33 million times [25]. Despite this high number, a systematic review conducted in Australia found only 6 “high quality” smoking cessation smartphone apps [26]. None of these 6 identified apps specifically addressed nondaily smoking. A recent Cochrane review identified 5 randomized controlled trials (RCTs) on smoking cessation smartphone apps [27]. None of these tested apps addressed nondaily smoking.

To address this need, our team developed a smoking cessation smartphone app specifically for nondaily smokers, the “Smiling instead of Smoking” (SiS) app. Building on research that found that nondaily smokers did not view nicotine addiction as relevant to their efforts to quit but instead viewed themes related to a positive self-identity and wellness as more important [28], we built an app that focused on fostering the experience of positive emotions. This treatment approach was inspired by the development of positive psychotherapy for smoking cessation [29,30]. The app’s overall conceptual model, as described elsewhere [31,32], is based on Social Cognitive Theory [33], in which goal setting, self-efficacy, and knowledge are leveraged to support behavior change.

Our app development plan entailed a staged process consisting of 3 studies. In study 1, we tested feasibility and acceptability in a local sample of nondaily smokers [31]. In this study, nondaily smokers interacted with study staff in in-person meetings, both during onboarding to the app and 3 weeks after the prescribed app use period ended. The feedback gained from the study participants was used to make modifications to the app. In study 2, the feasibility and acceptability of version 2 of the app were tested, this time in a completely remote setting with nondaily smokers recruited nationwide [34]. Again, insights gained during this study were used to modify the app. In both studies, app use was high and sustained throughout the treatment period. Participants rated the app favorably in terms of its ease of use and usefulness. Longitudinal surveys demonstrated increases in the hypothesized direction for the underlying mechanisms of behavior change (ie, increased confidence, decreased urge to smoke, and less positive perceptions of smoking). Long-term self-reported abstinence rates were high (ie, 53% and 56% of the participants reported 30-day point prevalence abstinence at the 6-month follow-up in studies 1 and 2, respectively) [31,34]. These data supported the investment in a randomized trial.

### Objectives

This study is study 3, which is a proof-of-concept RCT designed to test whether the SiS app successfully affects an early marker of smoking cessation success, namely, self-efficacy to abstain from smoking. We chose self-efficacy as our primary outcome variable as it is a causal component in many health behavior change theories and has been shown to be the primary mediator of conferring benefit in another mobile health (mHealth) intervention for smoking cessation, an SMS text messaging intervention [35]. The primary aim is to test for differences between randomized groups on the primary outcome, self-efficacy to remain abstinent, as measured at the end of treatment using the Smoking Self-Efficacy Questionnaire (SEQ-12) [36]. Self-efficacy to remain abstinent is hypothesized to be greater among participants in the SiS condition compared with 2 different but clinically relevant control conditions: the National Cancer Institute (NCI) smartphone app QuitGuide and the NCI smoking cessation brochure “Clearing the Air.” The QuitGuide app was chosen as a comparator as it is a widely used, publicly available smoking cessation app that is part of the best-practice recommendations for treating smokers in the health care setting. Thus, it is a credible control for using a smoking cessation smartphone app. It controls for time, attention, and modality of delivery of smoking cessation support. Clearing the Air was chosen as an additional comparator as, to date, there is no evidence to demonstrate that QuitGuide confers smoking cessation benefits. Although widely used and endorsed, QuitGuide has never been tested against a treatment-as-usual (TAU) control condition. Thus, by including the Clearing the Air group in the design, this RCT tests whether app support provides better smoking cessation support than the standard of care and, if so, whether the SiS app does so more effectively than the QuitGuide app. The secondary aim is to test for differences between randomized groups on secondary outcomes pertaining to treatment acceptability; treatment feasibility; and, in an exploratory way, treatment effectiveness in terms of smoking cessation outcomes. Also measured were the impacts on secondary proof-of-concept efficacy markers: positive affect, desire to smoke, and attitudes toward smoking.

## Methods

### Overall Trial Design

**Figure 1:**
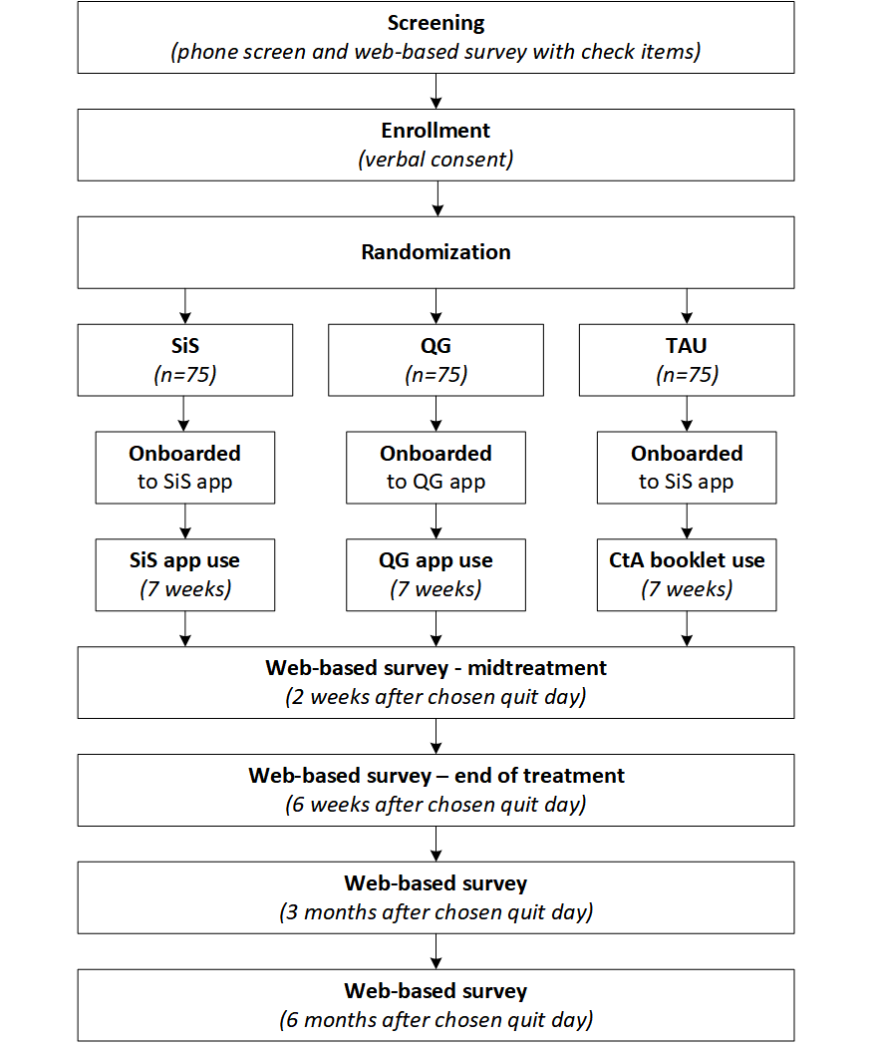
Flowchart of the trial protocol. CtA: Clearing the Air; QG: QuitGuide; SiS: Smiling instead of Smoking; TAU: treatment as usual.

Figure 1 shows the flowchart of this trial. This trial is a 3-group, randomized, fully remote, nationwide study. The trial aims to assess the efficacy of the SiS app version 3 in increasing smoking cessation self-efficacy in nondaily smokers compared with 2 control conditions representing current standards of care for providing low-barrier smoking cessation support. Participants in all treatment conditions will be asked to undertake a quit attempt while using smoking cessation support materials for a period of 7 weeks—1 week before and 6 weeks following their chosen quit date. Participants will be randomized to be onboarded to 1 of 3 conditions: the SiS smartphone app version 3, the NCI smartphone app QuitGuide, or the NCI brochure “Clearing the Air.” The onboarding process is an approximately 15-minute–long scripted phone call during which study staff will guide participants through how to use SiS, QuitGuide, or Clearing the Air. The script is time-matched across conditions. In all 3 groups, the script covers setting the quit date, logging cigarettes, identifying triggers and strategies for those triggers, and managing challenging times. The onboarding process will conclude with study staff instructing participants to use SiS, QuitGuide, or Clearing the Air for the next 7 weeks.

Follow-up assessments are conducted via web-based surveys administered via REDCap (Research Electronic Data Capture; Vanderbilt University) [37] 2, 6, 12, and 24 weeks after the chosen quit date. Survey completion is remunerated at up to US $25 for the 2-, 12-, and 24-week survey (US $10 if check items are responded to incorrectly; these items are interspersed throughout the survey and ask participants to use the survey interface to indicate specific answers, eg, “Please set the slider to ‘0’ to indicate ‘not at all confident’”) and US $50 for the 6-week survey, which is longer than the other surveys. The primary end point for this proof-of-concept RCT is end of treatment (ie, 6-week follow-up). The primary outcome is self-efficacy to remain abstinent from smoking, as measured by the SEQ-12 [36]. Secondary outcomes are shown in Table 1.

**Table 1:** List of measure outcomes and their assessment schedule.

Type of measure and specific measure				Week of study								
				0		2		6		12		24
**Primary outcome**												
	SEQ-12^a^			✓		✓		✓		✓		✓
**Secondary outcomes**												
	**Treatment acceptability**											
		Satisfaction with smoking cessation support (CSQ-8^b^)					✓					
		SUS^c^ (SiS^d^ or QG^e^ only)					✓					
		App likeability and satisfaction (SiS or QG only)					✓					
	**Treatment feasibility**											
		Self-reported amount of time spent applying content					✓		✓		✓	
		Use of smoking cessation strategies during treatment period					✓					
		Use of positive psychology strategies during treatment period					✓					
		Perceived impact of SiS, QG, or TAU^f^ on quitting					✓					
		Actual app use—continuously recorded (SiS or QG only)	✓		✓		✓		✓		✓	
		Self-reported app use—weeks 0 to 2 (SiS or QG only)			✓							
		Self-reported app use—weeks 3 to 6 (SiS or QG only)					✓					
	**Exploratory treatment effectiveness outcomes**											
		Self-reported 30-day point prevalence abstinence	✓		✓		✓		✓		✓	
		Cigarette reduction	✓		✓		✓		✓		✓	
**Exploratory outcomes (ie, secondary proof-of-concept efficacy outcomes)**												
	**Positive affect**											
		PANAS^g^	✓		✓		✓		✓		✓	
	**Desire to smoke**											
		Brief QSU^h^	✓		✓		✓		✓		✓	
	**Attitudes toward smoking**											
		ATS^i^	✓		✓		✓		✓		✓	
		DCB^j^-Short Form	✓		✓		✓		✓		✓	

^a^SEQ-12: Smoking Self-Efficacy Questionnaire.

^b^CSQ-8: Client Satisfaction Questionnaire.

^c^SUS: System Usability Scale.

^d^SiS: Smiling instead of Smoking.

^e^QG: QuitGuide.

^f^TAU: treatment as usual.

^g^PANAS: Positive and Negative Affect Schedule.

^h^QSU: Questionnaire of Smoking Urges.

^i^ATS: Attitudes Toward Smoking Scale.

^j^DCB: Decisional Balance Inventory for Smoking.

### Ethics Approval

This protocol is consistent with the CONSORT-EHEALTH (Consolidated Standards of Reporting Trials of Electronic and Mobile Health Applications and Online Telehealth) checklist [38]. This study was registered with ClinicalTrials.gov (NCT04672239) and approved by the Mass General Brigham Institutional Review Board (protocol 2020P003466).

### Trial Participants

Participants who meet all inclusion criteria and pass the screening process will be included in the trial. The eligibility criteria are (1) age of ≥18 years, (2) smartphone ownership (Android or iPhone only), (3) current nondaily smokers who smoke at least weekly and no more than 25 out of the past 30 days, (4) lifetime history of having smoked ≥100 cigarettes, (5) willingness to make a smoking quit attempt as part of this study, and (6) currently residing in the United States. The screening process entails a screening phone call with study staff during which the eligibility criteria are assessed, followed by a web-based survey containing check items that test that screening participants are able to complete web-based surveys. Incorrectly completed check items render a participant ineligible for the study. Under unusual circumstances (eg, a participant reports a technical glitch to the study team), the study team will review the survey to evaluate the time spent on the survey and consistency of survey responses across phone-screen answers and the survey and across measures and, if consistent, may proceed with enrollment of the participant. Participants will also be required to provide contact information for 1 to 2 contacts who can assist study staff in locating participants for follow-up surveys. After screening, study staff will ask screening participants to choose their smoking cessation quit date. Study staff will then schedule the study enrollment call to occur 8 days before this date. Consent is obtained verbally during the enrollment phone call after nondaily smoking status is once again confirmed. A total of 225 nondaily smokers will be enrolled and randomized. To ensure adequate representation of racial and ethnic minorities, we will close enrollment to non-Hispanic White participants when 54.2% (122/225) of the planned total sample size have been enrolled as, nationwide, 54% of nondaily smokers are non-Hispanic White as per the 2014 National Health Interview Survey [39].

### Randomization

Eligible participants will be randomly allocated in a 1:1:1 ratio to the 3 treatment conditions: SiS, QuitGuide, and Clearing the Air. Randomization is done using the PROC PLAN procedure in the SAS statistical software package (SAS Institute) with a block size of 4.

### Treatment and Control Conditions

Participants in all treatment conditions are instructed to use the provided smoking cessation materials (ie, SiS, QuitGuide, or Clearing the Air) for 7 weeks starting on the day of their onboarding. They are also informed that they may use any additional smoking cessation support that they would like to use during this time.

#### Treatment Condition: SiS Smartphone App

Participants randomized to the treatment condition will use version 3 of the SiS smartphone app. We will first describe the app in its current form and then describe the ways in which version 3 differs from version 2. Adjustments that were made in transitioning from versions 1 to 2 are detailed in the study 2 outcome paper [34].

**Figure 2:**
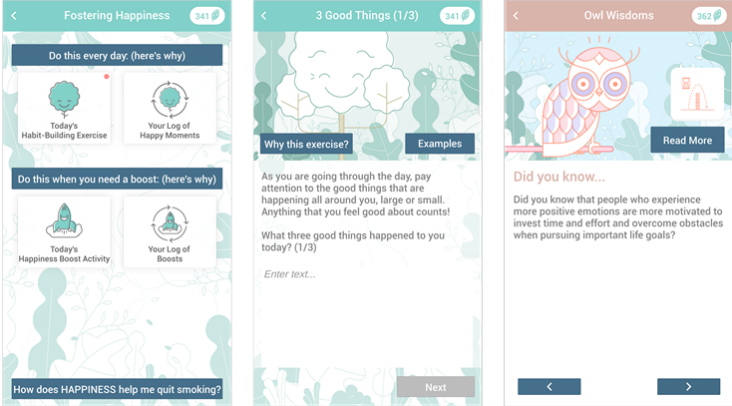
Screenshots of Smiling instead of Smoking version 3. The app prompts users to complete 1 happiness habit-building exercise every day and try out a happiness-boost activity, as needed. Information is provided throughout the app to explain the relevance of the positive psychology activities, including through a science-oriented feature called “Owl Wisdoms”.

**Figure 3:**
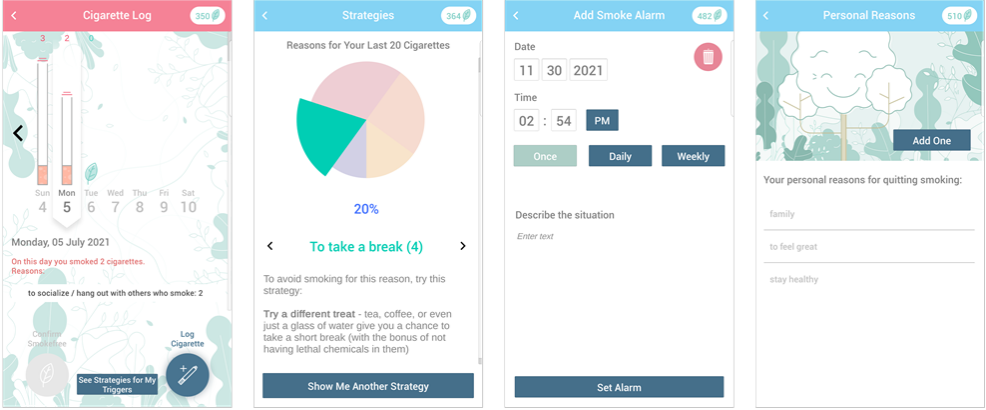
Screenshots from Smiling instead of Smoking version 3. While engaging in positive psychology content, app users are also prompted to complete smoking cessation activities within the app, including logging their cigarettes, which generates feedback on strategies for their triggers, and setting reminders to stay smoke-free, which reminds participants of their own personal reasons for quitting smoking.

The SiS app is a smartphone app that engages participants in positive psychology exercises (Figure 2) and smoking cessation activities (Figure 3). It is part of an emerging generation of smoking cessation smartphone apps that provide smoking cessation tools within a specific therapeutic approach (eg, acceptance and commitment therapy [25,40]). In our case, the focus is on maintaining positive affect. Reduced positive affect during smoking cessation is a common experience of substantial magnitude [41]. The use of the SiS app seeks to offset this effect as experiencing positive affect during the process of smoking cessation is related to increased self-efficacy to abstain from smoking [42], decreased desire to smoke [43,44], and greater readiness to process self-relevant health information [45]. Engaging in positive psychology exercises is also pleasurable and, thus, may be a particularly effective way to keep users engaged with the app [31,34].

Each day, participants are asked to complete a positive psychology habit-building exercise. The goal of completing these exercises is for participants to develop a habit of noticing and savoring the positive experiences they encounter in everyday life. The app selects 1 of 3 exercises each day at random (ie, “3 Good Things,” “Rose, Thorn and Bud,” and “Savoring,” as tested in prior work) [46]. If they have not completed this exercise by 7 PM, the app sends a push notification with a reminder to do so. App users are also encouraged to engage in a happiness-boost activity throughout the day as needed. The goal of the boost activity is to serve as a “rescue” activity during moments of craving or particularly low positive affect. Completing these activities is meant to provide a momentary boost in positive affect. To provide variety, the app randomly selects 1 of 11 possible happiness-boost activities each day as derived from the positive psychology literature. This differentiation between habit-building and boost activities was introduced in version 3. In version 2, there was a total of 5 happiness exercises. In study 2, participants commented that they would like to have more variety. Thus, in version 3, we limited the daily exercises to the 3 activities most suitable for habit-building and offered more variety through the introduction of boost activities. Throughout the app, information is provided to explain why participants are asked to complete positive psychology activities while quitting smoking, including through a feature called “Owl Wisdoms,” which are push notifications that provide participants with relevant findings from the literature.

The smoking cessation content of the app is presented through the use of a number of app tools (Figure 3). The tools and information are in line with clinical practice guidelines for smoking cessation (ie, United States Clinical Practice Guidelines) [47] and are presented in a way that conforms to published recommendations for smoking cessation apps [48]. Each day, participants are asked to log their cigarettes or confirm that they were smoke-free. When participants log cigarettes, they are asked to indicate their reason for smoking by choosing from a list of common triggers or writing in their own response. After participants log cigarettes, they have access to a pie chart that offers personalized strategies matching their smoking triggers. Every 2 to 3 days, participants receive a push notification to complete a specific activity within the app that focuses on smoking cessation, called a “Behavioral Challenge.” The timing of the “Behavioral Challenges” is anchored on the resettable quit date that participants specify in the app so as to provide participants with temporally appropriate smoking cessation activities as they progress. These “Behavioral Challenges” guide participants through the app’s smoking cessation tools, which enable setting reminders, note keeping (ie, personal reasons for quitting), and using distractions to get through moments of craving (ie, a game called “Magma Bear”) and provide information on the benefits of quitting smoking. For example, 5 days before their chosen quit date, participants receive a push notification to write down their personal reasons for quitting smoking; 2 days before the chosen quit date, they are asked to look at the pie chart of their triggers for smoking.

**Figure 4:**
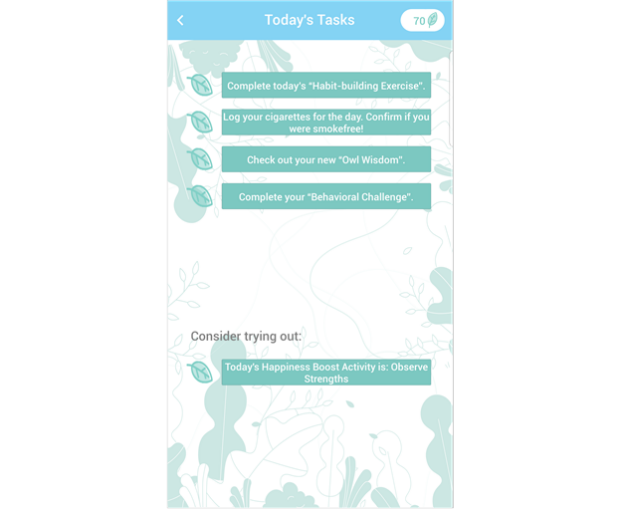
Screenshots from Smiling instead of Smoking (SiS) version 3. The “Today’s Tasks” tool was added in version 3 of the SiS app to enhance prescriptive clarity.

To make it easy for participants to remember what they should do on a given day, the SiS app version 3 provides an overview of all tasks required that day in a feature called “Today’s Tasks” (Figure 4). This feature was added based on secondary data analyses of app use data collected during the SiS study 2 [49]. Results indicated that participants completed the assigned tasks with a relatively high completion rate (57% of behavioral challenges and 45% of daily happiness exercises). Tasks that were offered but not required were rarely completed (on <7 days). These findings suggested to us that prescriptive clarity may enhance app engagement and, thus, in SiS version 3, we incorporated a tool that enhances prescriptive clarity.

#### Control Condition 1: NCI “QuitGuide” Smartphone App

Participants randomized to this control condition will use the NCI “QuitGuide” smartphone app. This smartphone app is 1 of 2 smartphone apps that the NCI currently has publicly available for use on their Smokefree.gov website. Referral to this website is recommended for treating smokers in health care settings [50]. QuitGuide is frequently used as a comparison app in smartphone app smoking cessation studies [40,51-55]. Although QuitGuide was not developed specifically for nondaily smokers, it targets both nondaily and daily smokers (ie, when downloading the app, users are asked to specify their smoking pattern, which includes both nondaily and daily patterns of smoking). Some SiS features have direct analogs in QuitGuide; others do not. The onboarding script leads QuitGuide users through the QuitGuide content in the same order as for SiS to the degree possible—setting the quit date (SiS and QuitGuide), happiness-promoting activities versus tracking mood and craving (SiS vs QuitGuide, respectively), guidance on quitting (via behavioral challenges in SiS vs “Learn to Quit” information in QuitGuide), logging cigarettes (SiS and QuitGuide), strategies for smoking triggers (pie chart in SiS and various graphs in QuitGuide), setting reminders (time-based in SiS and time- and location-based in QuitGuide), and as-needed tools (happiness-boost activities and suggested strategies in SiS vs tips in response to reporting craving or negative affect in QuitGuide). This control condition controls for time, attention, and modality of delivery of smoking cessation support.

#### Control Condition 2: NCI “Clearing the Air” Brochure

Participants randomized to this control condition will use the NCI booklet “Clearing the Air” [56]. This booklet has been used as a “treatment as usual” comparison condition in past RCTs evaluating phone-based mHealth smoking cessation technologies (ie, SMS text messaging). It remains a standard-of-care smoking cessation treatment today. Similar to SiS and QuitGuide, it is a low-barrier option for offering smoking cessation support. Unlike SiS and QuitGuide, it offers this support in a widely accepted booklet format rather than via mobile technology. During the scripted onboarding call, study staff will lead participants through the booklet’s content in the same order as implemented in the SiS behavioral challenges (ie, reflecting on reasons to quit, informing about the benefits of quitting, logging smoking and its triggers, and formulating strategies for dealing with triggers). By comparing with this control group, it can be tested whether the SiS app provides an advantage over the currently existing standard of care. This is an important question for implementation.

### Measures

#### Primary Outcome Measure

Although, ultimately, the goal of engaging with a smoking cessation smartphone app is smoking cessation, we chose a process indicator toward that goal as the primary outcome of this proof-of-concept RCT. Namely, we selected self-efficacy to abstain from smoking as a process indicator of subsequent smoking cessation. Self-efficacy has been identified as the primary mediator through which an SMS text messaging intervention conferred smoking cessation benefits. Similar to our study, the latter trial used the “Clearing the Air” booklet as the control condition and showed that self-efficacy observed 1 month after the chosen quit date mediated group differences in smoking cessation at the 6-month follow-up [35]. In this study, self-efficacy to abstain from smoking will be measured using the SEQ-12 [36], a 2D 12-item self-report scale measuring a person’s confidence in their ability to abstain from smoking when facing internal stimuli (eg, feeling depressed) and external stimuli (eg, being with smokers) on a 0 to 100 slider scale (ie, 0=*not at all confident that I can refrain* to 100=*extremely confident that I can refrain*). Scale scores are created by averaging across items and range from 0 to 100. Higher scores indicate greater self-efficacy to abstain from smoking.

#### Secondary Outcome Measures

This is the first randomized trial to evaluate the SiS app. As this is a proof-of-concept trial, our secondary aims cover several domains: treatment acceptability; treatment feasibility; and, in an exploratory way, treatment efficacy in terms of smoking cessation outcomes.

##### Treatment Acceptability

As an overall measure of treatment acceptability, we will assess participants’ satisfaction with the smoking cessation support they will receive as part of the study. To this end, we will use the widely used Client Satisfaction Questionnaire. [57]. For this study, and with permission, we adjusted the wording of the introduction to this scale to clarify that the items refer to the smoking cessation support received as part of participating in this study. The wording of the 8 items will be used verbatim (eg, “How satisfied are you with the amount of help you received?”). Scores are summed across items and range from 8 to 32, with higher scores indicating greater satisfaction. For the 2 randomized groups using an app (SiS and QuitGuide), we will also assess the satisfaction with the apps from a technical standpoint. To this end, we will use the 10-item System Usability Scale, [58], as used in other studies on smoking cessation smartphone apps [59,60]. This 10-item scale provides a global view of subjective assessments of usability. To make clear that we are seeking feedback on the app and not on other aspects of the study, we have replaced the phrase “this system” (original scale) with “app” (modified scale; eg, “I found the app unnecessarily complex” 5-point Likert scale: 1=*strongly disagree* to 5=*strongly agree*). Scale scores range from 0 to 100, with greater scores indicating a more favorable perception of the usability of the app. To obtain an overall summary impression combining technical issues and smoking cessation support satisfaction, the survey will also include 2 stand-alone items: “How satisfied are you with the smoking cessation support this app provided you with?” (5-point Likert scale: 1=*very unsatisfied*, 2=*somewhat dissatisfied*, 3=*neither satisfied nor dissatisfied*, 4=*somewhat satisfied*, and 5=*very satisfied*) and “How much did you like using the smoking cessation app we asked you to use?” (5-point Likert scale: 1=*I strongly disliked using the app*, 2=*I somewhat disliked using the app*, 3=*I neither liked nor disliked using the app*, 4=*I somewhat liked using the app*, and 5=*I strongly liked using the app*). These scales and items will be assessed only once, at end of treatment (week 6).

##### Treatment Feasibility

To assess treatment feasibility, we will assess processes that are relevant across all 3 randomized groups and issues that are specific to app use. For all 3 groups, the end-of-treatment survey (week 6) will include scales to assess the degree to which participants applied the smoking cessation content delivered during treatment and the degree to which they practiced the type of positive psychology habits taught in the SiS app. Our hypothesis is that participants randomized to SiS will use taught smoking cessation strategies at least comparably with those in the QuitGuide and Clearing the Air groups and will use positive psychology strategies more frequently than QuitGuide and Clearing the Air participants, who are not taught these skills. We will assess the use of smoking cessation strategies using a list of 19 items developed for another randomized trial on smoking cessation [30]. Participants rate their agreement with statements about the support they received (eg, “I used the Smiling Instead of Smoking app/QuitGuide app/Clearing the Air pamphlet [logic-branched by treatment group] the way I was supposed to (in the days leading up to and following my quit attempt)”) and the things they did while quitting (eg, “I avoided situations that would make me want to smoke”). Items are rated on a 5-point Likert scale (1=*strongly disagree* to 5=*strongly agree*). Higher scores indicate greater use of smoking cessation strategies. This scale also included items that referred to the positive psychology content of the intervention (eg, “I told myself that quitting smoking would eventually improve my moods, not make them worse” and “I focused on the good things that happened each day”). To assess the use of positive psychology techniques more directly, we will also use the Appreciation Scale [61]. This 18-item scale assesses the frequency with which one engages in thoughts and actions to heighten awareness of the blessings in one’s life (eg, “I enjoy the little things around me like the trees, the wind, animals, sounds, light, etc.,” rated on a 7-point frequency scale: 1=never to 7=more than once a day) and the degree to which one feels appreciative (eg, “I recognize and acknowledge the positive value and meaning of events in my life,” rated on a 7-point agreement scale: 1=strongly disagree to 7=strongly agree). The score is created by sum scoring, with higher scores indicating a more appreciative mindset. As a subjective evaluation of the received treatment, participants will be administered 17 items to assess the impact of the treatment they received. The stem of the items is “The ‘Smiling Instead of Smoking app, QuitGuide app, or Clearing the Air pamphlet [logic-branched by treatment group]...” which is followed by possible impacts (eg, “...gave me confidence that I can quit smoking,” “...made me think it was worthwhile for me to quit,” and “...helped me stay positive while quitting”). The same items were used to evaluate version 2 of the SiS app [34]. These scales and items will be assessed only once, at end of treatment (week 6). Finally, 1 treatment feasibility item will be administered in multiple surveys (weeks 6, 12, and 24). This item seeks to assess the degree to which participants engaged with the provided smoking cessation materials: “*During this past week, how much time did you spend applying or contemplating the content of the SiS app, QG app, or ‘Clearing the Air’*?” (in minutes).

Regarding app use (SiS and QuitGuide groups only), passive data recording will be used that time-stamps each action participants take in the app. For QuitGuide, we will receive these data from the Smokefree.gov team. From these data, we will calculate the number of days the participants used the app during the treatment period. As objective reality and subjective experience can differ, the surveys will also ask participants to self-report app use. At week 2, they will be asked to report the number of days they used the app during weeks 0, 1, and 2. At week 6, they will be asked to report the number of days they used the app during weeks 3, 4, 5, and 6. In addition, they will be asked to self-report how many minutes per day they used the app.

##### Exploratory Treatment Effectiveness Outcomes

All surveys will include items to assess self-reported 30-day point prevalence abstinence. Participants will be asked the following—“what is your current smoking status?”—with the following options: “I smoke daily*,*” *“I smoke non-daily (and have smoked in the past 7 days),” “I smoke non-daily (but have NOT smoked in the past 7 days),”* and *“I do not smoke at all.”* If a participant reports abstinence, they will then be asked, “have you been abstinent during the past 30 days?” (yes or no). If a participant does not report abstinence, they will be asked to indicate how many cigarettes they smoked over the past 7 days. From these data, we will code a binary indicator for self-reported 30-day point prevalence abstinence (yes or no) and calculate an indicator of cigarette reduction by subtracting the total number of cigarettes smoked in the week before baseline from the total number of cigarettes smoked in the past week.

#### Exploratory Outcomes (ie, Secondary Proof-of-concept Efficacy Outcomes)

##### Overview

As our secondary outcomes spanned several domains, including acceptability, feasibility, and exploratory estimates of gold-standard effectiveness markers, we designated secondary proof-of-concept efficacy outcomes as exploratory outcomes. Similar to our primary outcome, these outcomes measure constructs that, according to our conceptual model, should be affected by treatment and, in turn, should affect long-term smoking cessation. All these measures are self-report measures and are included in every survey. As proof-of-concept process markers, the primary end point of interest is the end of treatment. Treatment effects are hypothesized to have occurred at that point, with the hope that these impacts translate into subsequent smoking cessation success.

##### Positive Affect

The SiS app is designed to offset expected decreases in positive affect as smokers undertake a quit attempt [41]. Thus, if successful, positive affect should be higher among participants randomized to the SiS group by treatment end. To measure positive affect, we will use the 20-item Positive and Negative Affect Schedule [62]. Participants are given a list of 20 adjectives (eg, interested or distressed) and asked to rate to what extent they have experienced these states over the past week. Rating options range from 1 to 5 (1=*very slightly or not at all* to 5=*extremely*). A total of 10 items measure positive affect (ie, attentive, interested, alert, excited, enthusiastic, inspired, proud, determined, strong, and active), and the other 10 items measure negative affect. Positive affect items are sum scored to create the positive affect score, with a higher number reflecting a more frequent experience of positive affect.

##### Desire to Smoke

Previous research has indicated that experiencing positive affect during the process of smoking cessation is related to a decreased desire to smoke [43,44]. Although nondaily smokers are not nicotine-dependent, craving, or, rather, the desire to smoke, is the most common reason why nondaily smokers attempting to quit relapse to smoking during the 2 weeks after their chosen quit date [63]. Thus, reducing the desire to smoke is a meaningful outcome in supporting nondaily smokers with quitting. To measure the desire to smoke, we will use the 10-item Brief Questionnaire of Smoking Urges [64]. Participants rate items about their desire to smoke (eg, “A cigarette would taste good now*”* and “*I could control things better right now if I could smoke”*) on a 7-point Likert scale (1=*strongly disagree* to 7=*strongly agree*). Items are sum scored to create the total score, with higher scores indicating a greater desire to smoke.

##### Attitudes Toward Smoking

Experiencing positive affect during the process of smoking cessation is related to greater readiness to process self-relevant health information, particularly information about the negative health effects of smoking [45]. In our previous studies on the SiS app, we conceptualized that this readiness would entail shifts in the appraisals of the pros and cons of smoking [31,34]. In these single-group studies, we found that positive appraisals of smoking decreased, as expected, but that negative appraisals stayed largely the same. In this study, we will measure these constructs in the same way, assessing both the participants’ expectations of the effect of smoking on them using the Attitudes Toward Smoking Scale (ATS-18) [65] and the importance of these expectations on their decision to smoke using the Decisional Balance Inventory for Smoking (DCB)-Short Form [66]. The ATS-18 consists of 18 items that participants rate on a 5-point Likert scale (*1=strongly disagree* to *5=strongly agree*). These items address the adverse effects (eg, “Smoking is extremely dangerous to my health”), psychoactive benefits (eg, “A cigarette calms me down when I am stressed”), and pleasures (eg, “It feels so good to smoke”) of smoking. The DCB consists of 6 items rated on a 0 to 100 slider anchored at 0=*not at all important* and 100=*extremely important* and asks participants the following: “HOW IMPORTANT is each statement to your decision to smoke RIGHT NOW?” Both the pros (eg, “Smoking cigarettes relieves tension”) and cons (eg, “I’m embarrassed to have to smoke”) of smoking are represented. For both the ATS-18 and DCB, items are sum scored to create subscale scores, with higher scores indicating greater endorsement.

#### Contextual Variables

At baseline, participants will be asked if they have ever tried to quit smoking (yes or no). If yes, they will be asked to indicate the number of previous quit attempts and what type of support they have used to quit smoking before (ie, group counseling, one-on-one counseling, quitline, web-based chat with a tobacco cessation specialist, website, social networking site for smoking cessation, SMS text message program, smoking cessation app, and medications). If they have previously used an app, they will be asked to indicate which one. In subsequent surveys, they will be asked which of these methods they have used since their onboarding call.

For descriptive purposes, the baseline survey will also include items on participants’ demographics, past mental health diagnoses, smoking history, nicotine dependence (Fagerström Test for Nicotine Dependence), previous quit attempts, generalized self-efficacy (Generalized Self-Efficacy Scale), and personality (Single-Item Measures of Personality). Across time, also for descriptive purposes, participants will answer questions about their e-cigarette use, alcohol use, depression (Center for Epidemiological Studies Depression-10), generalized anxiety (Generalized Anxiety Disorder-7), capacity to experience pleasure (Snaith-Hamilton Pleasure Scale), optimism (Life Orientation Test-Revised, State Optimism Measure, and Brief State Optimism Measure), and coping style (Brief Coping Orientation to Problems Experienced).

### Masking or Blinding

This proof-of-concept trial is not blinded. The same staff member who onboards participants to their smoking cessation materials may be the staff member reaching out via phone and email to remind participants to complete the surveys. However, contact with study staff is minimal and scripted. The initial survey invitations are automated using REDCap. Only surveys that are not completed within 3 days of the automated survey invitation will trigger a reminder email from the study staff, using an email template, and a phone call.

### Sample Size

To select the sample size for this proof-of-concept trial, we were guided by one of the few mHealth smoking cessation studies that examined processes of smoking cessation over time. That study tested the effectiveness of an SMS text messaging intervention and, similarly to our study, used the “Clearing the Air” booklet as the control condition [67]. The study identified self-efficacy as the primary mediator of the observed salutary effect of the intervention on smoking cessation. Specifically, self-efficacy observed 1 month after the chosen quit date mediated group differences in smoking cessation at the 6-month follow-up [35]. The observed group difference between treatment and control at 1 month after the quit date on self-efficacy was Cohen *d*=0.66. Thus, we conservatively powered our proof-of-concept RCT to detect an effect size of Cohen *d*=0.50. As no trials have been published that test the effectiveness of QuitGuide, we assumed that QuitGuide would be as effective as Clearing the Air and that SiS would be more effective than either of them. Using SAS PROC POWER, we calculated that we would need a sample size of 64 per group to detect an effect of Cohen *d*≥0.50. Assuming a retention rate of 85%, we would need to enroll 75 participants per group to retain 64 by end of treatment. Thus, the total number of participants to be enrolled in this trial is 225 (3 randomized groups×75 participants to be enrolled in each group=225).

### Analysis Plan

All successfully enrolled participants (ie, those who passed the screening process and were successfully onboarded to the assigned smoking cessation materials) will be included in the outcome analyses. Each follow-up survey will include 5 check items randomly interspersed throughout the survey. If a participant incorrectly responds to more than one of the 5 check items, this participant’s entire survey for this time point will be considered unreliable and excluded from the analyses. If a participant incorrectly responds to only 1 of the 5 check items, the participant’s data for the scale in which the error occurred will be considered unreliable and excluded from the analyses. All analyses will be conducted using SAS (version 9.4).

In this proof-of-concept RCT, the primary outcome is self-efficacy to abstain from smoking, as measured by the SEQ-12. The primary end point is end of treatment, which occurs 6 weeks after the originally chosen quit date. To test for differences between randomized groups, we will fit a fixed-effects repeated-measure model using PROC MIXED where observations are modeled as nested within persons. The predictors included in the model will be group (ie, SiS vs QuitGuide vs TAU), time (ie, baseline, week 2, week 6, week 12, and week 24), and the group×time interaction effect. A contrast statement will be used to derive the test statistic for the group comparison at week 6, our primary end point, given the overall longitudinal model. This contrast statement will test the null hypothesis that SiS=QuitGuide=TAU. If this null hypothesis is rejected (*P*<.05), we will conduct pairwise follow-up tests that compare the 3 possible pairwise comparisons (ie, SiS vs QuitGuide, SiS vs TAU, and QuitGuide vs TAU). Subscale scores can be computed for the SEQ-12 (ie, confidence to abstain from smoking in response to internal vs external triggers). For this trial, we consider the total SEQ-12 score as the primary outcome. We will also report outcomes on subscale scores.

The secondary outcomes of this proof-of-concept RCT are exploratory. Thus, we will not correct for multiple testing. We will fit 1 model per secondary outcome using an α of .05 for all analyses. To test for differences between randomized groups for outcomes that are assessed multiple times (ie, time spent applying content, app use, self-reported 30-day point prevalence abstinence, and cigarette reduction), our overall modeling approach will be the same as that for the primary outcome variable. That is, for each outcome, we will fit a fixed-effects repeated-measures model where observations are modeled as nested within persons. The predictors included in the model will be group, time, and the group×time interaction effect. However, the definition of the time effect varies across these secondary outcomes based on their assessment schedule. The distribution of the outcome variables also varies across these variables. Thus, we will implement this overall modeling approach for these variables but adjust the model to match the assessment schedule and distribution of the variable. To test for differences between randomized groups for outcomes that are only assessed at end of treatment (ie, satisfaction with smoking cessation support, system usability, app likeability and satisfaction, use of smoking cessation strategies, use of positive psychology strategies, and perceived impact of treatment), for each outcome, we will use a fixed-effects model where group is the only predictor and the score at end of treatment is the dependent variable. Histograms will be used to guide the selection of the most appropriate distribution. If the group effect is statistically significant (*P*<.05), we will conduct pairwise follow-up tests that compare the 3 possible pairwise comparisons (ie, SiS vs QuitGuide, SiS vs TAU, and QuitGuide vs TAU) using the Tukey adjustment. These follow-up analyses will not be necessary for secondary outcomes that are only assessed for the SiS and QuitGuide conditions.

For exploratory outcomes (ie, secondary proof-of-concept efficacy outcomes), we will use the same statistical approach as for the primary outcome but will match the analysis to the distribution of the variable under examination. For example, 30-day point prevalence abstinence will be a binary variable. Thus, we will use a binomial distribution with a logit link function.

## Results

Recruitment began in January 2021 and ended June 2022 at this time. The final follow-up occured in January 2023. Specifically, the last partial follow-up response was collected on January 4, 2023, and efforts to obtain the rest of these participants' data ceased on January 6, 2023. We expect to publish the main outcomes of this study in summer 2023.

## Discussion

This RCT will provide the first test of the SiS app versus relevant comparison conditions—the NCI smoking cessation brochure “Clearing the Air” and the NCI smoking cessation smartphone app QuitGuide. The primary outcome will be self-efficacy to remain abstinent, as measured using the SEQ-12. Secondary outcomes include treatment acceptability; treatment feasibility; proof-of-concept efficacy markers such as positive affect, desire to smoke, and attitudes toward smoking; and, in an exploratory way, treatment effectiveness in terms of smoking cessation outcomes. We hypothesize that participants using the SiS app will have higher self-efficacy to abstain from smoking than participants in either of the control conditions as the SiS app is specifically designed for nondaily smokers, whereas the other 2 comparison conditions are geared more generally toward daily smokers. In terms of acceptability and feasibility, we anticipate that participants using the SiS app will find the treatment at least as acceptable and feasible as the comparison conditions (ie, not worse). We further hypothesize that we may be able to detect differences in proof-of-concept efficacy markers as well as smoking cessation outcomes such that participants using the SiS app will have higher levels of positive affect, a lower desire to smoke, reduced prosmoking attitudes (eg, lower ratings of the psychoactive benefits of smoking, lower ratings of the pleasures of smoking, and lower ratings of the pros of smoking), and a greater self-reported 30-day point prevalence abstinence proportion at the end of treatment compared with both comparison conditions.

This study is innovative in several ways. First, the study results will indicate whether a fully powered test of the effectiveness of the SiS app is warranted. This is a vital contribution to future clinical trials. Currently, nondaily smoking is not addressed by clinical practice guidelines for smoking cessation and, thus, treatment recommendations for this increasingly prevalent pattern of smoking are not clear. Therefore, testing whether this novel treatment may warrant a larger, fully powered trial moves the field forward toward the critically important goal of providing empirically based smoking cessation support for nondaily smokers. This is especially important as other efforts to identify effective treatments for this group of smokers have so far been unsuccessful. Specifically, in recent years, nicotine replacement therapies have been tested, but studies have failed to show efficacy [68,69]. The SiS app uses a different treatment modality (mHealth rather than medication) and targets a different mechanism (bolstering positive affect rather than ameliorating withdrawal) and, thus, is a novel, promising treatment approach. If our study provides evidence that the SiS app promotes greater self-efficacy to abstain from smoking than the comparison treatments and if the study gives a positive indication of treatment acceptability and feasibility, we plan to submit a proposal for a fully powered RCT to test the efficacy of the SiS app in promoting smoking abstinence in nondaily smokers.

The results of this study will also be useful more generally to understand how people who smoke interact with smoking cessation self-help materials and how a smartphone app may confer benefits. This study includes numerous measures that provide insights into treatment acceptability and feasibility, both from the point of view of the user and based on observed behaviors that are passively recorded. The chosen measures address a diversity of aspects that are relevant to treatment development (eg, does the app successfully teach or prompt the use of smoking cessation behaviors? Does the app successfully engage users in positive affect–supporting behaviors?) and implementation (eg, do people like the treatment? Will they adhere to the treatment instructions? Do they perceive the treatment to be impactful?). To date, only a handful of studies have looked at these important indexes, and even fewer have done so in a randomized design.

A limitation of this study is the lack of biochemical verification of smoking status for entry into the study and to verify smoking abstinence at the end of treatment. At study entry, we will use several check items and consistency checks to evaluate whether interested participants are providing attentive, consistent, and detailed information about their current smoking behavior. This will increase the likelihood that participants entering the study are nondaily smokers. The lack of biochemical verification of smoking abstinence at end of treatment may inflate our estimates of smoking abstinence overall, but we do not expect that it will bias the comparison between treatment groups. In total, 2 strengths of the study are the efforts to recruit a diverse, representative sample of nondaily smokers and the use of 2 clinically relevant control groups that allow for the answering of different but equally important questions. With respect to recruitment, our efforts to ensure that we enroll a proportion of ethnic and racial minority participants that is representative of nationwide estimates will strengthen the generalizability of our results. With respect to the comparison groups we chose, in most health care settings, referral to smoking cessation apps is not provided. Although many such apps exist, only a handful have been tested empirically and, thus, the evidence base to support such a standard-of-care referral is slim. In these settings, providing written materials to support smoking cessation, such as the NCI booklet “Clearing the Air,” remains a standard of care. Thus, comparing the SiS app with that control group answers the following question: would it be useful to change the standard of care to include referral to an app? This is an important practical question that can guide decision-making in health care settings. The other control group addresses the following scientifically relevant question: is the SiS app better than existing smartphone apps for smoking cessation? Although the NCI QuitGuide app has never been tested in a randomized trial designed to test its effectiveness, it is a widely used smartphone app and a recommended resource. Thus, it is an important benchmark that can help tease apart the effects of using a credible, well-designed app from the treatment mechanisms targeted and implemented in the SiS app. Note that this design also represents the first true test of QuitGuide against an established standard of care (ie, referral to “Clearing the Air”). Comparing QuitGuide to Clearing the Air provides insight into whether referral to this publicly available and widely used app is indeed beneficial to nondaily smokers.

Thus, we believe that this study will provide data to inform the next steps toward providing smoking cessation support for nondaily smokers while also providing important, multifaceted insights into smoking cessation smartphone app treatment development and implementation.

## Appendix

Multimedia Appendix 1 Peer-review report by Mentored Research Scholar Grant - American Cancer Society.

## Abbreviations

ATS-18: Attitudes Toward Smoking Scale
CONSORT-EHEALTH: Consolidated Standards of Reporting Trials of Electronic and Mobile Health Applications and Online Telehealth
DCB: Decisional Balance Inventory for Smoking
mHealth: mobile health
NCI: National Cancer Institute
RCT: randomized controlled trial
REDCap: Research Electronic Data Capture
SEQ-12: Smoking Self-Efficacy Questionnaire
SiS: Smiling instead of Smoking
TAU: treatment-as-usual

## Data Availability

The data sets generated during this study will be available from the corresponding author upon reasonable request.
