# Supplementary material for: Testing the Outcomes of a Smoking Cessation Smartphone App for Nondaily Smokers: Protocol for a Proof-of-concept Randomized Controlled Trial
Source: JMIR Res Protoc. 2023 Feb 14;12:e40867. doi: 10.2196/40867 (PMC9975937; doi:10.2196/40867)
Supplement: Multimedia Appendix 1 [file resprot_v12i1e40867_app1.pdf]

March 2, 2017

To: Postdoctoral Fellowship, Mentored Research Scholar Grant, Research Scholar Grant, and Research Scholar Grant - Insurance Applicants

From: Elvan C. Daniels, MD, MPH
Scientific Program Director, Cancer Control and Prevention Research

Re: Critiques

Please see the critique of your application at the end of this memorandum. This is to provide you with additional information concerning the peer review process, and to answer some frequently asked questions.

1. Each application is assigned to at least two committee members, who submit their critiques before they attend the Peer Review Committee meeting. The critiques are made available to all members of the committee before the proposal is discussed. Critiques can be revised by the reviewers in light of discussions at the open meeting. Due to practical time constraints, not all applications are orally discussed during the meeting of the Peer Review Committee.

2. The terms used to describe the proposals are: **Outstanding, Excellent, Good, Fair and Non-Competitive** in decreasing order of enthusiasm. When the proposal is discussed in the open meeting or in online threaded discussions, the reviewers present their critiques to the entire committee. If the opinions of the primary and secondary reviewers are very different, the committee strives to ensure that all of the important issues are clarified to the full committee prior to voting. This can result in a preliminary review being "talked up" or "talked down" so that the eventual priority score may occasionally seem somewhat inconsistent with one of the reviews. Each member of the committee, including stakeholders, scores the proposal, and the final ranking is based on the average of approximately twenty individual scores. We do not provide scores to the applicants, because we believe that it is much more useful to focus on the overall rating and recommendations of the peer review committee rather than the numeric score.

3. The Council will carry out the final review of the proposal at its meeting in mid-March 2017 to determine the paylines for each Peer Review Committee. No additional, unsolicited information can be accepted concerning the applications unless requested by the Program Director.

4. We allow one resubmission of Postdoctoral Fellowships and two for Research Scholar Grants, Mentored Research Scholar Grants, and Pilot and Exploratory Studies, but applicants are urged to consult the ACS policies on eligibility (available at www.cancer.org) before doing so. Applicants are encouraged to read the critiques carefully and to thoughtfully consider the comments and suggestions of the reviewers. Following review of the critiques, applicants are encouraged to schedule time to speak with their Program Director who can provide additional insights about the review of the application by the peer review committee. Below are instructions on how to schedule a phone call with the Program Director.

The American Cancer Society uses scheduling software that will allow you to directly schedule a conference call with the Program Director during the months of March and April to discuss the critiques of your grant application. This system saves a great deal of time for you and for our staff in arranging the many calls between applicants and the program directors. Using this tool, you will be able to see the available times on my schedule and sign up for an appointment time. In addition, if a change needs to be made, you can log back into the site and adjust the scheduled time on the calendar.

PLEASE NOTE:
Due to the high application load this cycle and limited amount of time available for the scheduling of calls, if you do not plan or are not eligible to resubmit on April 1, please schedule calls in April and reserve time in March for those who plan to resubmit for the Spring 2017 cycle. If you find that the schedule is full or you are unable to find a suitable time, please contact Chanda Felton (Chanda.felton@cancer.org) and she will arrange a call.

If, for any reason, this system does not work properly for you, please email Annette Jordan (annette.jordan@cancer.org) for help with the program.

Instructions:

1. Go to https://www.appointmentquest.com/provider/2080155035
2. Select the Make Appointment for Grant Application Follow-Up box
3. Select Ellie Daniels as the Program Director
4. Check Availability
5. Choose a time and Make Appointment
6. Complete the new Customer Profile
7. Fill in the Committee Code for application cycle *(i.e., CPPB, CPHPS, PCSM)*
8. You will receive an automated email with the time and day chosen for the call

Please note that all information will be purged from the system at the end of April so that no personal information is ever made available to an outside organization.

RESEARCH SCHOLAR GRANT CRITIQUE TEMPLATE

Application ID #:	130323	Reviewer Number (1 or 2)	1
Applicant Name	Hoeppner Bettina		
Project Title	**Positive Psychology Smoking Cessation Smartphone App for Nondaily Smokers**		

PART I CANDIDATE:

Dr. Hoeppner received her doctorate in Psychology in 2007 from the University of Rhode Island and did a post-doc in Addictions at Brown University 2007-10. She also holds a Masters in Statistics from U Rhode Island. She has been an Assistant Professor in the Department of Psychiatry at Harvard Medical School since 2012. She has strong editorial board experience given her short time as faculty and has 46 publications, with 14 as first author. She completed a K award (EMA and advanced stats as well as understanding mechanisms of change in college students during quit attempt) in early 2016 and a local (MGH) award during which she developed version 1 of the app discussed in this application. She is also PI on a currently internally-funded project that has overlapping methodology with the proposed project – collecting real time quitting experience (EMA) data from intermittent smokers. She has received numerous awards in the past few years, including a scholarship to NIH's mobile Health Summer Institute.

PART II RESEARCH PLAN: *(Reply to previous reviews, if applicable*)

Response to Review: This is a second revision. The applicant was very responsive to the first critique, addressing all issues. The most recent review only had one suggestion – to provide support for an institutional colleague (Dr. Park) who was listed as an unpaid consultant so as to ensure sufficient time for this very experienced expert to participate in this project. In this submission, Dr. Park has been provided with 5% salary support and is noted to have a more active role in the project, especially the qualitative data analyses.

Overview: This is a well written and thought-out proposal to refine an existing Version 1 smartphone app (developed with internal award) to deliver a smoking cessation intervention based on positive psychology to non-daily smokers motivated to quit and test it in an RCT. In the proposed project, feedback from non-daily smokers will be solicited to further develop the app, first by gathering structured user feedback in a small local sample of non-daily smokers (n=30) to guide the design of Version 2.0, and then by crowd-sourcing feedback from the larger community of non-daily smokers nation-wide (n=90) via on-line recruitment/data collection (with a subsample of 20 participating in virtual focus groups) to further refine and adapt Version 2.0 to create Version 3.0. A proof-of-concept randomized controlled trial (RCT) (n=226) to test the app's effectiveness in enhancing self-reported short-term (6-week) abstinence rates, and its impact on the theorized mechanisms of change (via EMA data collection) will be conducted, using Facebook for recruitment. In all three phases, participants will be asked to use the app for at least three weeks, one week before and two weeks after their quit attempt, and to complete surveys at enrollment, 2 weeks, 6 weeks, 3 months and 6 months post quit. The treatment group of the proof-of-concept RCT will be asked to self-monitor smoking and related thoughts and feelings using an ecological momentary assessment (EMA) approach during the first three weeks of app use to inform knowledge of the real-time process of smoking cessation in non-daily smokers. The study is powered for outcomes at 6 weeks but will examine them (although

underpowered) at 3 and 6 months. The applicant has a strong team with appropriate expertise for this project. As a relatively young investigator, Dr. Hoeppner has included a senior mentor on her team and a co-investigator with qualitative analyses expertise. She has an existing relationship with the app developer and has an outside consultant with expertise in on-line recruitment.

Research Plan:

Significance: Intermittent (non-daily) smokers represent 22% of current smokers - an increasing proportion of smokers, a disproportionate proportion being minorities. These intermittent smokers are subject to the same health sequealae as daily smokers - some at lesser rates (cancers) while others at similar rates (heart disease). Current treatments and theoretical models of cigarette smoking are designed for daily smokers and may not be appropriate for intermittent smokers. This subpopulation's high rate of failure in quit attempts suggests a need for targeted smoking cessation interventions, which are lacking. This proposal aims to identify appropriate strategies and a scalable intervention for this group (smartphone app), using positive psychology, as evidence suggests this may be an efficacious methodology.

Cancer Relevance: Reduce smoking among an increasing population. The cancer risk of non-daily smoking is substantial (40-50% of that seen in daily smokers).

Innovation: 1. Enhancing positive affect states to support smoking cessation in non-daily smokers – few interventions address non-daily smokers specifically and this approach is uniquely addressing reasons nondaily smokers smoke; 2. Using smartphone app to deliver treatment; 3. Uses EMA data to refine theory and understanding; 4. Uses social media to recruitment and online processes to engage, follow and collect data; 5. Will have a free, available product at end of study for dissemination (if warranted by positive findings).

Team: Strong team, including two more senior mentors (Drs. Kelly and Park, each 5%), Statistician and graduate research assistant (part time) and outside consultant/collaborators with whom she has some existing relationships.

Letters of Support are specific and detailed, as appropriate and enthusiastic.

Approach: Specific aims: (Aim 1) to develop the smartphone app by soliciting detailed feedback from non-daily smokers in two pilot studies (n=30 structured user feedback and n=90 online data collection and online focus groups in n=20 subsample); (Aim 2) to conduct a proof-of-concept randomized controlled trial (RCT) to test the effectiveness of the proposed app to enhance short-term abstinence rates (n=226); (Aim 3) to enhance theory through real-time data (EMA) analysis of non-daily smokers undergoing a quit attempt.

Study design: Participants across studies, excepting participants in the RCT control group (Clearing the air by NCI), are instructed to use the app for 3 weeks (1 week before, 2 weeks following the quit attempt), and optionally thereafter and complete surveys at enrollment, 2-week, 6-week, 3-month, and 6-month follow-up. Some specific data, such as ecological momentary assessment (EMA) data (Pilot #1, tx group of the RCT), structured user feedback (Pilot #1), and focus groups (Pilot #2), will vary across studies. Stratified recruitment plan to ensure nationally representative sample (based on 2014 NHIS demographics – e.g., 54% non-Hispanic Whites)

Environment: Appropriate for proposed work

Budget $788,639 over 4 years. Budget appropriate for scope of work – although difficult to see where outside consultants are funded given the budget structure.

Other Support: no issues

PART II. USE OF HUMAN OR ANIMAL SUBJECTS

Protection of Human Subjects: low risk and potential benefits significantly outweigh risks – informed consent procedures articulated for each study

Inclusion of Women, Minorities and Children. Good, children 18-20 only, which is appropriate

PART III – RECOMMENDATIONS

OVERALL RECOMMENDATIONS:

Enthusiasm for this proposal is extremely high as it has potential for high impact among a growing group of smokers not currently being addressed.

Strengths of this proposal include:

- Uses a national online sample for RCT with stratified recruitment to ensure national representativeness which should result in strong generalizability
- Has theoretical mechanism of change for design and testing
- End user input into refining app at two levels
- Strong design and sufficiently powered for proof-of-concept RCT (at 6 weeks only)
- Addresses an area that is emerging and understudied and addresses disparities (disproportionate minority representation among intermittent smokers)
- Very well detailed and articulated methodology
- Strong team with appropriate expertise
- Builds upon previous work so much of infrastructure is already extant (version 1)
- Promises to provide immediate free access if found successful.

RESEARCH SCHOLAR GRANT CRITIQUE TEMPLATE

Application ID #:	130323	Reviewer Number (1 or 2)	2
Applicant Name	Hoeppner		
Project Title	**Positive Psychology Smoking Cessation Smartphone App for Nondaily Smokers**		

ABSTRACT:

This second resubmission addresses non-daily smoking (22% of smokers), an increasingly prevalent smoking pattern that has been increasing over the last two decades, that carries a significantly increased cancer risk compared to non-smokers. Existing treatments are ill-suited for non-daily smoking, because they are based on nicotine dependence, and do not appeal to non-dependent non-daily smokers. Treatments that target the unique characteristics of non-daily smoking are sorely needed. This application proposes to develop a smartphone app that acts as a behavioral, in-the-pocket, coach and uses positive psychology exercises to enhance quitting success. Positive psychology exercises will be used to enhance and/or maintain happiness, which will stimulate non-daily smokers to enact healthier alternatives to smoking by increasing self-efficacy, and decreasing desire to smoke and defensiveness about smoking-related health information. The investigative team proposes to work closely with non-daily smokers to refine the app using an existing fully-functional Version 1.0 as a starting point, and collect ecological momentary assessment (EMA) data to enhance understanding of the process of smoking cessation in non-daily smokers generally. The project has three phases: (1) in-depth interviews with 30 local non-daily smokers to develop Version 2.0; (2) solicit crowd-sourcing feedback from 90 online non-daily smokers to develop Version 3.0, including five focus groups in a subsample of 20 participants, who are willing to discuss their experience and suggestions in a video conference call, and (3) test the effectiveness of Version 3.0 of the app to improve abstinence rates 6 weeks after the chosen quit day in a randomized trial with 226 non-daily smokers recruited online.

PART I **CANDIDATE**: PI is well qualified to carry out the proposed study. Great track record of publications in smoking cessation and substance abuse research methodology.

PART II **RESEARCH PLAN:** *(Reply to previous reviews, if applicable*)

Significance: non-daily smoking is a significant issue. Thus group of smokers is an important group, as they have significant cancer risk, and may be low hanging fruit with regard to smoking cessation. This mobile application, if successful is highly scalable, and therefore has the potential for high public health impact.

Cancer relevance – highly relevant to cancer, given the risk associated with smoking

Innovation - novel mobile technology intervention in a novel population. Also novel methodology of staged testing, from small usability study that includes feedback and EMA data from smokers to crowd sourcing using online resources to randomized trial.

Approach – several strengths: version 1.0 of intervention is already developed. Small qualitative study is appropriate for usability, and use of EMA is novel and informative in this context. In the final stage, there is a RCT for proof of concept. Sample size and analytic plan seems appropriate for research question.

The resubmission addresses the concern about lack of attention to the qualitative process. The team has added Dr. Elyse Park, a qualitative expert

Research team: PI has experience is smoking cessation research, and is teaming up with the CBITS group at Northwestern, who is a leader in mhealth research and development

PART III BUDGET AND JUSTIFICATION OF BUDGET:

Reasonable and justified

PART IV USE OF HUMAN OR ANIMAL SUBJECTS:

Risks and protections are acceptable and adequate

PART V OVERALL RECOMMENDATIONS:

This is an outstanding application that has the potential for high impact.
